# Association of academic performance of premedical students to satisfaction and engagement in a short training program: a cross sectional study presenting gender differences

**DOI:** 10.1186/1756-0500-7-105

**Published:** 2014-02-24

**Authors:** Jean Joel R Bigna, Loic Fonkoue, Manuela Francette F Tchatcho, Christelle N Dongmo, Dorothée M Soh, Joseph Lin Lewis N Um, Paule Sandra D Sime, Landry A Affana, Albert Ruben N Woum, Steve Raoul N Noumegni, Alphonce Tabekou, Arlette M Wanke, Herman Rhais K Taffe, Miriette Linda N Tchoukouan, Kevin O Anyope, Stephane Brice E Ella, Berny Vanessa T Mouaha, Edgar Y Kenne, Ulrich Igor K Mbessoh, Adrienne Y Tchapmi, Donald F Tene, Steve S Voufouo, Stephanie M Zogo, Linda P Nouebissi, Kevine F Satcho, Wati Joel T Tchoumo, Moise Fabrice Basso, Bertrand Daryl N Tcheutchoua, Ako A Agbor

**Affiliations:** 1 Faculty of Medicine and Biomedical Sciences, University of Yaoundé 1, P.O. Box 1364, Yaoundé, Cameroon; 2 Goulfey District Hospital, P.O. Box 62, Kousséri, Goulfey, Cameroon; 3 Department of Biology, CEMPLEX premedical training center, Yaoundé, Cameroon; 4 Faculty of Medicine, Pharmacy and Odonto-Stomatology, Cheikh Anta Diop University, Dakar, Senegal; 5 Faculty of Medicine and Pharmaceutical Sciences, University of Douala, Douala, Cameroon; 6 Higher Institute of Medical Technologies, Yaoundé, Cameroon

**Keywords:** Academic engagement, School performance, Health study, Medical education, Medical preparation, Africa, Medical student, Satisfaction, Training school

## Abstract

**Background:**

It is important that students have a high academic engagement and satisfaction in order to have good academic achievement. No study measures association of these elements in a short training program. This study aimed to measure the correlation between academic achievement, satisfaction and engagement dimensions in a short training program among premedical students.

**Methods:**

We carried out a cross sectional study, in August 2013, at *Cercle d’Etudiants, Ingénieurs, Médecins et Professeurs de Lycée pour le Triomphe de l’Excellence* (CEMPLEX) training center, a center which prepares students for the national common entrance examination into medical schools in Cameroon. We included all students attending this training center during last examination period. They were asked to fill out a questionnaire on paper. Academic engagement was measured using three dimensions: vigor, dedication and absorption. Satisfaction to lessons, for each learning subject was collected. Academic achievement was calculated using mean of the score of all learning subjects affected with their coefficient. Pearson coefficient (r) and multiple regression models were used to measure association. A *p* value < 0.05 was statistically significant.

**Results:**

In total, 180 students were analyzed. In univariate linear analysis, we found correlation with academic achievement for vigor (r = 0.338, *p* = 0.006) and dedication (r = 0.287, *p* = 0.021) only in male students. In multiple regression linear analysis, academic engagement and satisfaction were correlated to academic achievement only in male students (R^2^ = 0.159, *p* = 0.035). No correlation was found in female students and in all students. The independent variables (vigor, dedication, absorption and satisfaction) explained 6.8-24.3% of the variance of academic achievement.

**Conclusion:**

It is only in male students that academic engagement and satisfaction to lessons are correlated to academic achievement in this short training program for premedical students and this correlation is weak.

## Background

In Cameroon, the infatuation for medical studies is high; about 6000-8000 students coming from high school, write the entrance examination into medical school and only 10–15% of them are retained [1]. The training period of students who have the advanced level General certificate of education from high school is important to succeed the national common entrance examination into medical school and can be influenced by several factors. Among these determinants, it is known that academic engagement influences academic achievement among medical students [2]. Academic engagement is an indicator that combines academic identification and academic participation of students [3]. Engagement of students in the university is related to each student and the context in which he/she is learning [4]. Maintaining an optimal engagement of students is important because a low academic engagement is a risk factor for academic failure [5,6]. Several methods exist to measure engagement [7]. In this study, we focused on students’ active participation, satisfaction and emotional commitment to their learning. It is important to note that students were satisfied to the teaching method. Teaching institute can play a key role on engagement and satisfaction and should consider what it is that motivates students to become engaged and should use these findings to improve student engagement with university work [8,9].

Studies have demonstrated that engagement is positively correlated to more active learning and student’s participation [10-17]. Some studies conducted previously, have demonstrated correlation between academic engagement and academic achievement [2,5,18,19]. In these studies, one was conducted among medical students [2]. The impact of satisfaction of students to teaching was not measured and no study was conducted among students in short training programs. The short training program can better reflect the association between academic achievement and academic engagement, and satisfaction because engagement may fluctuate in long term studies. This study aimed to measure the association between academic achievement, satisfaction and engagement dimensions in premedical student in short training program. Our hypothesis was that more engaged and more satisfied premedical students would be more likely to have better academic success.

## Methods

### Design and study setting

We carried out a cross-sectional study at a center which trains and prepares students for the common competitive national entrance examination into medical schools. The name of this training center is *Cercle des Etudiants, Ingénieurs, Médecins et Professeurs de Lycée pour le Triomphe de l’Excellence* (CEMPLEX). This training center was situated in Yaoundé, Cameroon. We enrolled all students attending this training center in August 2013. This center prepares students coming from high school for the competitive entrance examination into medical school.

### Participants and recruitment

We used a convenience sample with the goal to introduce all students in the study (n = 225). The participation was proposed to all students two days before introduction to the study. Participation and response rates were 100% on the day of data collection. We removed 48 (21.3%) participants due to incomplete data.

### Academic achievement

The content for the preparation was given in French for ten weeks from July to August 2013. The subjects involved during the preparation at CEMPLEX were the same subjects written at the national competitive common entrance examination into medical schools in Cameroon i.e: Biology, Physics, Chemistry, Logic, Language, and General Knowledge. We have applied the same method of evaluation and scores calculation in national competitive common entrance examination. The exam is done in two parts: first part (major part with a coefficient 4 allocated to this part) and second part (minor part with a coefficient 1 allocated to this part). The first part contains the first three learning subjects and the duration is three hours and the second part contains the others subjects with a total duration of one hour. We collected scores from final exam. The form of evaluation used was multiple choice questions (MCQ). Table 1 presents MCQ distribution and coefficient of each learning subject. Each learning subject was scored from 0 to 100. The general score was calculated with the following formula considering coefficient of each learning subjects:

$$\frac{\left(2*\text{Biology}\right)+\text{Chemistry}+\text{Physics}+\left(\frac{1}{3}\right)*\left(\mathrm{Logic}+\text{English}+\text{General}\;\text{culture}\right)\;}{500}$$

**Table 1:** MCQ distribution and coefficient of each learning subject

**Exam part**	**Subject**	**Coefficient (% of quotation)**	**Number of MCQ (% of MCQ)**
Part 1 (major part)	Biology	2 (40.0)	50 (31.3)
	Chemistry	1 (20.0)	25 (15.6)
	Physics	1 (20.0)	25 (15.6)
Part 2 (minor part)	Logic	1/3 (6.7)	20 (12.5)
	Language	1/3 (6.7)	20 (12.5)
	General culture	1/3 (6.7)	20 (12.5)

MCQ = multiple choice questions.

### Utretch work engagement scale for students (UWES-S)

The measurement of academic engagement was determined by using the UWES-S. The version used has 14 items, featuring scores ranging from 0 “Never” to 6 “Always” as in the study of Casuso-Holgado et *al.*[2]. Table 2 presents items of UWES-S. The score of each item was after take back to a notation from 0 to 100. An exploratory factorial analysis of the scale was conducted. Kaiser-Meyer-Olkin measure was 0.784 and Bartlett’s test 0.000. The method of varimax rotation showed a three-dimensional structure (vigor, dedication and absorption) explaining 52.0% of the total variance. The reliability of each dimension demonstrated an acceptable internal consistency, low compared to previous studies [2,20], with Cronbach’s coefficients of 0.63, 0.36 and 0.65 respectively for vigor, dedication and absorption. The UWES-S was presented in French language.

**Table 2:** Statements in each item measure of engagement dimensions

**Response number**	**Statement**
**Vigor**	
V1	When I’m studying, I feel mentally strong
V2	I can continue for a very long time when I am studying
V3	When I study, I feel like I am bursting with energy
V4	When studying, I feel strong and vigorous
V5	When I get up in the morning, I feel like going to class
**Dedication**	
D1	I find my studies to be full of meaning and purpose
D2	My studies inspire me
D3	I am enthusiastic about my studies
D4	I am proud of my studies
D5	I find my studies challenging
**Absorption**	
A1	Time flies when I’m studying
A2	When I am studying, I forget everything else around me
A3	I feel happy when I am studying intensively
A4	I can get carried away by my studies

### Satisfaction to lessons

Satisfaction was collected for each of six learning subjects on scale from 0 “No satisfied” to 3 “Completely satisfied”. We used the following question: “Are you satisfied with the quality of teaching in (learning subject)?” The score of each item was after take back to a notation from 0 to 100. The reliability demonstrated an acceptable internal consistency, with Cronbach’s coefficient of 0.72.

### Statistical analysis

Data was coded, entered, and analyzed using the Statistical Package for Social Science (SPSS) version 20.0 for Windows (SPSS, Chicago, Illinois, USA). We described continuous variables using means with standard deviations, and categorical variables using their frequencies and percentages. The main analysis was guided towards a search for the correlations between engagement dimensions and academic achievement. We used Pearson’s linear correlation to search for this correlation. We used simple and multiple regression models. The T-test was used to compare continuous variables and the χ^2^ test or its equivalents to compare categorical variables. A p-value less than 0.05 was considered statistically significant. We dichotomized academic result and therefore compared students with good academic result (general score ≥ 50/100) and students with bad academic result (general score < 50).

### Ethical considerations

The study was approved by National Research Ethics Committee for Human Health of Cameroon. Written informed consent was obtained from the students and his/her parent or guardian in accordance with the Helsinki Declaration of World Medical Association [21]. We collected all data in the same day during the last exam.

## Results

In total, 180 students were analyzed. Sex ratio male/female was 1/2. Table 3 presents general characteristics of study population. Male students were more aged than female (p = 0.043). Female students were more satisfied with teaching than male (p = 0.013). There was no statistical difference between male and female students as regards the academic achievement and engagement dimensions. In univariate linear analysis, vigor (r = 0.338; *p* = 0.006) and dedication (r = 0.287; *p* = 0.021) were correlated to academic result only in male students (Table 4). In multiple regression linear analysis, association of engagement and satisfaction were lowly correlated to academic result (R^2^ = 0.159; *p* = 0.035), due to vigor only and in male students only (Table 5). In this multivariate linear regression analysis, the independent variables (vigor, dedication, absorption and satisfaction) could explain 6.8-24.3% of the variance of academic achievement. Fourteen (7.8%) students had good academic results. The only variable which predicts good academic results was dedication (*p* = 0.021) (Table 6). Vigor, dedication, absorption and satisfaction were all correlated among themselves (Table 7).

**Table 3:** General characteristics of study population

	**Female**	**Male**	***p***	**All**
**n**	**116**	**64**		**180**
Age, years (SD)	18.6 (1.7)	19.2 (1.8)	.043	18.8 (1.8)
Academic achievement, scale 0 to 100 (SD)	34.7 (8.6)	36.1 (9.7)	.321	35.2 (9.0)
Satisfaction to lessons, scale 0 to 100 (SD)	62.4 (17.0)	56.1 (14.3)	.013	60.2 (16.3)
Engagement dimensions, scale 0 to 100 (SD)				
Vigor	57.3 (14.8)	56.1 (12.8)	.575	56.9 (14.1)
Dedication	83.7 (15.3)	80.8 (13.3)	.213	82.7 (14.6)
Absorption	65.2 (18.3)	63.5 (17.8)	.551	64.6 (18.1)

SD = standard deviation.

**Table 4:** **Pearson correlation coefficient (r [ *p *-value]) between engagement dimensions, satisfaction to lessons and academic result**

**Predictor variables**	**All students**	**Female**	**Male**
**n**	**180**	**116**	**64**
Vigor	.046 (.543)	-0.106 (.258)	.338 (.006)
Dedication	.083 (.268)	-0.014 (.880)	.287 (.021)
Absorption	.070 (.353)	.108 (.250)	.017 (.896)
Satisfaction	.067 (.371)	.110 (.241)	.033 (.797)

**Table 5:** Multiple regression linear analysis of engagement dimensions and satisfaction to lessons as predictor variables for academic result

**Predictor variables**	**All students**	**Female**	**Male**
	**Partial correlation coefficient (p-value)**		
Vigor	-0.006 (.946)	-0.204 (.054)	.327 (.028)
Dedication	.061 (.457)	-0.043 (.671)	.184 (.183)
Absorption	.046 (.589)	.196 (.066)	-0.132 (.313)
Satisfaction	.050 (.524)	.133 (.160)	-0.082 (.521)
	**R**^**2 **^**(p-value)**		
	.011 (.734)	.058 (.154)	.159 (.035)

**Table 6:** Comparison of students with and without good academic result

	**Score ≥ 50**	**Score < 50**	***p***
	n = 14	n = 166	
Age, years (SD)	18.4 (1.7)	18.8 (1.8)	.342
Male, n (%)	7 (50.0)	57 (34.3)	.256
Satisfaction to lessons, scale 0 to 100 (SD)	59.5 (16.7)	60.2 (16.4)	.881
Engagement dimensions, scale 0 to 100 (SD)			
Vigor	61.0 (13.2)	56.4 (14.2)	.262
Dedication	89.8 (10.5)	82.1 (14.8)	.021
Absorption	66.7 (14.1)	64.4 (18.4)	.577

SD = standard deviation.

**Table 7:** **Pearson correlation coefficient (r [ *p *-value]) between vigor, dedication, absorption and satisfaction**

	**Vigor**	**Dedication**	**Absorption**
Dedication	.345 (< .001)		
Absorption	.417 (< .001)	.309 (< .001)	
Satisfaction	.226 (.002)	.193 (.009)	.149 (.046)

## Discussion

This is, to the best of our knowledge, the second study in which relation was assessed between academic achievement and academic engagement with 3 dimensions of UWES-S, the first been done by Casuso-Holgado et *al.*[2]. It is the first study conducted among premedical students attending short training program with the aim of writing the competitive entrance examination into medical school. Also, we assessed the association between academic achievement and satisfaction to lessons. For limitations, this is a cross-sectional study design and it is not the best method to assess causality between variables, but it is a help to guide future research.

This study analyzed the association between engagement dimensions, satisfaction and academic achievement in premedical students. After multiple analyses, significant associations were found in male students only concerning vigor and dedication. But these relationships were not sufficiently strong (r < 0.4) and only vigor was implicated in multiple regression analysis. Dedication was the only variable which may predict good academic result but the association was not linear in all students. Academic engagement and satisfaction to lessons were weakly correlated to academic achievement (R^2^ < 0.2). We have found a correlation between vigor, dedication, absorption and satisfaction.

The results obtained can permit us to deduce that vigor weakly and linearly influences academic achievement. This correlation was present only in male students (r = 0.33, p = 0.03). We also found that vigor was not a determinant for good academic result. In the study of Casuso-Holgado et *al.*[2] like in ours, vigor was the only variable which was significantly associated to academic achievement in multivariate analysis, but only in male students in our study. In others studies, there was also an association between vigor and academic results [18,19,22,23]. Dedication influenced academic achievement weakly, and in male students only, in univariate analysis. It had no effect on multivariate analysis with other engagement dimensions and satisfaction. But, dedication was the only determinant to have good academic result (*p* = 0.021). As regards absorption, it did not influence any academic achievement. Results concerning dedication and absorption were not consistent with previous studies [2,18,19,22-24]. In multivariate analysis, engagement dimensions associated to satisfaction were correlated to academic result like in others studies [2,18,19,22-24], but this correlation was weak (R^2^ < 0.2) and was found in male students only. In addition, vigor was the only variable that had a partial positive correlation in this multivariate analysis. These differences can be explained by several reasons. The duration of the training program was short (8 weeks) and therefore, engagement was not influenced by duration. With the ultra-competitiveness and high infatuation for medical study in Cameroon [1]: the students can be highly engaged but the school results don’t follow. The method of calculation of academic achievement was different in our study: we did not use grade point average, success rate and performance rate like other studies [2,24-29]. In our study, we also integrated satisfaction to lessons in multivariate analysis. And students were particularly followed and psychologically boosted in this training program with the goal to maintain high engagement and good satisfaction. Satisfaction was not correlated with academic achievement and it was different between male and female students. This result was explained by the fact that in this training program, students were particularly followed and female students were more receptive for psychologically boosted program. Other possible reasons could be that teachers perceived male students’ temperament and educational competence more negatively than female [30]. This can influence teachers to take more time to satisfy female students than male students.

Analyses have shown that the engagement of male students influenced academic performance but it is not the same for female students. This can be explained by the fact that, in a study where female students had obtained higher grades than male; learning in class, study habits and attitudes and peer relationships (with females having more narrow relationships with their peers, compared to males) were different between male and female students [31]. It would be necessary for future studies to understand why engagement and satisfaction not influence academic performance in female students.

In this study, engagement and satisfaction explained only 6.8-24.3% of the variance of academic achievement. In another study, engagement only explained 18.9-23.9% of the variance, consistent with our results [2]. The impact of independent variables was low to influence academic results, and the reasons are the same presented above.

With these results, it is necessary to evaluate in future studies in short training programs, other factors that can influence academic achievement. In this study, the correlation of engagement dimensions and satisfaction to lessons were weak and only in male students. Others factors and others methods to measure engagement and satisfaction were necessary to investigate. These factors can be personality traits [32,33] and emotional intelligence [32,34-37].

## Conclusion

We conclude that, in short training programs, engagement dimensions and satisfaction to lessons were weak correlated to academic achievement only for male students. This correlation which is weak in male students and totally absent in female students, suggest that others factors influence academic achievement of students in short training programs.

## Abbreviations

UWES-S: Utrecht work engagement scale for students; CEMPLEX: Cercle d’Etudiants, Ingénieurs, Médecins et Professeurs de Lycée pour le triomphe de l’Excellence.

## Competing interests

The authors declare that they have no competing interests.

## Authors’ contributions

JJRB conceived and designed the study; involved in the edition of data base; collected, analyzed and interpreted data; drafted and revised the manuscript. MFFT and CND designed the study, involved in the edition of data base, collected data, and critically revised and reviewed the manuscript. LF, DMS, JLLNU, PSDS, LAA, ARNW, SRNN, AT, AMW, HRKT, MLNT, KOA, SBEE, BVTM, EYK, UIKM, AYT, DFT, SSV, SMZ, LPN, KFS, WJTT, MFB, and BDNT designed the study, collected data, and critically revised and reviewed the manuscript. AAA designed the study, and critically revised and reviewed the manuscript. All authors approved the final version of the manuscript. All authors agreed to be accountable for all aspects of the work in ensuring that questions related to the accuracy or integrity of any part of the work are appropriately investigated and resolved.

## Authors’ information

JJRB (MD) is the General Manager Assistant and the Head Assistant of Biology department in CEMPLEX training center. LF (MD) is General Manager and the Head of Biology department in CEMPLEX training center. All others authors are medical students and assistant teachers of Biology in CEMPLEX training center.
